# Comparison of Flocculation Methods for Sodium Alginate and Characterization of Its Structure and Properties

**DOI:** 10.3390/foods14172970

**Published:** 2025-08-26

**Authors:** Yuxin Shi, Mingna Dong, Xuhui Lei, Zhiying Xu, Jiyan Sun, Yingying Zhao, Yichao Ma, Hui Zhou, Shu Liu, Yunhai He, Qiukuan Wang, Dandan Ren

**Affiliations:** 1 College of Food Science and Engineering, Dalian Ocean University, Dalian 116023, China; 2 National R&D Branch Center for Seaweed Processing, Dalian 116023, China; 3 Key Laboratory of Aquatic Product Processing and Utilization of Liaoning Province, Dalian 116023, China

**Keywords:** *Lessonia nigrescens*, sodium alginate, extraction process, structural analysis

## Abstract

This study investigated how different extraction parts of raw materials and different flocculation methods affect the extraction yield, structure, and properties of sodium alginate. The aim was to improve the quality of sodium alginate and provide theoretical guidance for upstream enterprises. In this study, *Lessonia nigrescens* (LN) was used as a raw material. The alkali treatment conditions were optimized. The optimal extraction conditions were determined to be a 2% sodium carbonate concentration, a duration of 4 h, a material-to-liquid ratio of 1:40, and a temperature of 60 °C, achieving an extraction yield of 43.03%. LN was categorized into blades, stipes, holdfasts, and whole seaweed for comparative analysis, and sodium alginate was flocculated using the acid, calcium, and ethanol methods. Structural and physicochemical analyses showed that the mannuronic acid/guluronic acid (M/G) ratios of the twelve sodium alginate samples ranged from 5.73 to 8.76. The LN part had a greater influence on the M/G ratio than the flocculation method. The relative molecular weight (2343–3074 kDa) and viscosity (170–331 mPa·s) exhibited consistent trends. For the same part, the effect of the flocculation method on the molecular weight followed the order ethanol > acid > calcium. The physicochemical properties of the extracted sodium alginate met the requirements specified in the physicochemical index standard GB 1886.243-2016 of China.

## 1. Introduction

**Figure 1:**
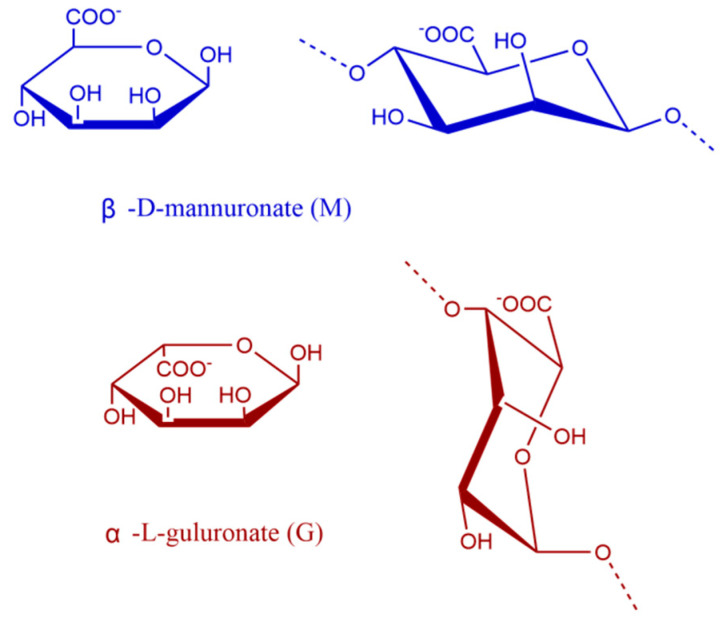
Representation of the conformation of the M and G monomers.

Brown algae, a group of large marine macroalgae found extensively across the global oceans, include commonly known species such as *Laminaria japonica*, *Undaria pinnatifida*, *Sargassum fusiforme*, and others. Brown algae are rich sources of various polysaccharides, including fucoidan, sodium alginate, agar, agarose, and carrageenan [1]. Sodium alginate extraction is an important way to utilize the resources of brown algae; it is a binary copolymer consisting of β-D-mannuronic acid (M) and α-L-guluronic acid (G) linked together by 1 → 4 glycosidic bonds in the order of homopolymer blocks (MM and GG blocks) and heteropolymer blocks (MG or GM blocks) (Figure 1) [2]. *Lessonia nigrescens* is a brown seaweed widely distributed along the South Pacific Chilean coast, where it grows rapidly and achieves high biomass levels in cold, nutrient-rich waters, making it an abundant resource [3]. It has high sodium alginate content and is commonly used as a raw material for alginate production.

Sodium alginate exhibits an excellent water-retaining capacity, thickening properties, and stability. These characteristics have contributed to its in high demand in biomedical, pharmaceutical, and bioengineering applications [4]. Sodium alginate is a nature-derived anionic polymer that initiates coagulation through contact activation, leading to blood clot formation. It activates thrombin, which converts fibrinogen into fibrin fibers. Therefore, sodium alginate is widely used in the medical industry [5]. Moreover, due to its biocompatibility, low cost, and film-forming ability, sodium alginate is widely used in the food preservation industry [6]. Commercially, alginate is mainly extracted from brown seaweed as soluble sodium alginate. The annual world production of brown algae in terms of dry weight is estimated to be approximately 85 thousand tons, from which about 23 thousand tons of alginate is obtained [7].

Since the commercial production of sodium alginate commenced in 1927, numerous scholars have investigated its extraction process. Although various extraction methods exist, they are all based on a common underlying principle. Specifically, under alkaline conditions, the insoluble alginate salts present in the algal cell wall matrix are transformed into water-soluble sodium alginate. This is followed by separation and purification to obtain the desired sodium alginate product [8].

Currently, the existing methods for the flocculation of sodium alginate include the acid method, calcium method, and ethanol method [9]. Enterprises predominantly utilize the calcium method due to its rapid extraction speed and the formation of larger precipitation particles. However, this method is associated with a low extraction rate and compromised product quality. Moreover, industrially produced sodium alginate often demonstrates poor storage stability, decreased viscosity, and a lower molecular weight. In recent years, the application of sodium alginate has seen a significant increase. Consequently, optimizing the flocculation method for sodium alginate holds significant importance in enhancing its quality, as well as improving the economic benefits for enterprises [10]. This optimization also contributes to advancing the development of the sodium alginate industry.

In this study, the alkaline treatment process was optimized using LN as the raw material. The optimal parameters were identified by systematically evaluating the influences of four key variables: the alkali concentration, treatment time, material-to-liquid ratio, and temperature. This evaluation involved designing both single-factor and orthogonal experiments. Sodium alginate samples were separated and extracted using acid, calcium, and ethanol methods. Their structural compositions and physicochemical properties were characterized accordingly. This study provides a reference basis for enterprises aiming to produce sodium alginate with diverse properties and applications.

## 2. Materials and Methods

### 2.1. Materials and Reagents

*Lessonia nigrescens*, sourced from Chile, South America, was purchased from Shandong Jiejing Group Co., Ltd. (Rizhao, China).

All chemicals were of analytical grade unless otherwise specified. D-Glucuronic acid, used in the meta-hydroxydiphenyl method, and commercial sodium alginate, used as the experimental control, were purchased from the Tianjin Damao Chemical Reagent Factory (Tianjin, China). Potassium bromide, required for Fourier transform infrared spectroscopy (FTIR), was obtained from the Tianjin Guangfu Fine Chemical Research Institute (Tianjin, China). 1-Phenyl-3-methyl-5-pyrazolone (PMP), used in high-performance liquid chromatography (HPLC), was provided by Aladdin Holding Group Co., Ltd. (Beijing, China). D-Mannuronic acid sodium salt and L-monoguluronic acid sodium salt (both chromatographically pure, HPLC ≥ 98%) were acquired from Qingdao Haihe Biotechnology Co., Ltd. (Qingdao, China). Acetonitrile (chromatographically pure) was sourced from Xilong Science Co., Ltd. (Shantou, China) and methanol (chromatographically pure) from Guangdong Guanghua Science and Technology Co., Ltd. (Guangzhou, China).

### 2.2. Instruments

The TMS-Pro texture analyzer was purchased from Beijing Ying Sheng Hengtai Technology Co., Ltd. (Beijing, China); the AL2D4 analytical balance and pH meter were provided by Mettler Toledo Instruments Co., Ltd. (Zurich, Switzerland); the 8S-1 magnetic stirrer was purchased from Shanghai Heying Instrument Co., Ltd. (Shanghai, China); the 101A-2ET electric blast drying oven was acquired from Shanghai Instrument Experimental Factory Co., Ltd. (Shanghai, China); the B-220 constant-temperature bath was supplied by Shanghai Yarong Biochemical Instrument Co., Ltd. (Shanghai, China); the enzyme labeling instrument was purchased from Boateng Instrument Co., Ltd. (VT, USA); the MX-S vortex oscillator was obtained from Shanghai Fuyao Trading Co., Ltd. (Shanghai, China); the KC-40A digital ultrasonic bath was provided by Jiekang Ultrasonic Equipment Co., Ltd. (Dongguan, China); and the NEXUS 2670 Fourier infrared spectrometer was purchased from PerkinElmer Co., Ltd. (Waltham, MA, USA).

### 2.3. Experimental Methods

#### 2.3.1. Optimization of the Alkaline Treatment Process of LN

##### Single-Factor Experiment on Alkaline Treatment Process

A single-factor experiment was conducted to investigate the sodium alginate alkaline treatment process, focusing on the effects of four key influencing factors: the alkali concentration, time, material-to-liquid ratio, and temperature [11,12]. Each factor was varied individually while maintaining the others at fixed levels to evaluate its effect on the extraction yield. The raw material LN was dried in an oven at 50 °C for 24 h, ground into a powder, and passed through a 50-mesh sieve. A total of 5.00 g of this powder was soaked in a 0.1 mol/L HCl solution at a material-to-liquid ratio of 1:30 (m/v) for 1 h, followed by filtration and washing three times with distilled water. Subsequently, the sample was mixed with sodium carbonate solutions at alkali concentrations of 1% m/v, 2% m/v, 3% m/v, and 4% m/v. These mixtures were prepared at varying material-to-liquid ratios of 1:30 g/mL, 1:40 g/mL, 1:50 g/mL, and 1:60 g/mL. The samples were subjected to alkaline treatment under different conditions, including varying treatment times (3 h, 4 h, 5 h, and 6 h) and temperatures (40 °C, 50 °C, 60°C, and 70 °C). Afterwards, the alginates were precipitated with 95% ethanol, filtered through gauze, and dried in an oven at 55 °C. The uronic acid content in the alkaline treatment solution was quantified using the meta-hydroxydiphenyl method [13].

##### Orthogonal Testing of Alkaline Treatment Processes

In this experiment, an L_9_(3^4^) orthogonal design was employed to examine the influences of four key factors on the extraction yield of sodium alginate from whole LN [14]. The selected factors were as follows: alkali concentration (Factor A), time (Factor B), material-to-liquid ratio (Factor C), and temperature (Factor D). The specific levels and parameter settings for these factors are detailed in Table 1. The whole seaweed raw material of LN was subjected to alkaline treatment according to the set conditions of each factor in Table 1. The extraction yield of sodium alginate under each condition combination was determined and analyzed.

#### 2.3.2. Effects of Different Flocculation Processes on the Extraction Yields of Sodium Alginate

The extraction of sodium alginate involved the conversion of water-insoluble salts of alginate from the algal cell wall matrix into water-soluble sodium alginate in an alkaline medium [15]. Following alkaline treatment, the sodium alginate solution was precipitated by the addition of ethanol, calcium chloride, or hydrochloric acid to obtain sodium alginate, calcium alginate, or alginic acid, respectively. These three methods are commonly referred to as the ethanol method, calcium method, and acid method [16,17,18].

In the acid method, 10% hydrochloric acid was added to the alkaline treatment solution at a volume ratio of 1:5 (*v*/*v*) of hydrochloric acid to alkaline treatment solution, followed by filtration. The alginate was subjected to liquid-phase conversion in an alkaline ethanol solution containing 95% ethanol and 4% sodium hydroxide for 40 min. Subsequently, the product was dehydrated with anhydrous ethanol to obtain sodium alginate, which was then dried in an oven at 55 °C and ground into a powder.

In contrast, in the calcium method, the pH of the alkaline treatment solution was adjusted to 7, and 10% calcium chloride solution was subsequently added at a 1:3 (*v*/*v*) ratio. After filtration, the sample was decalcified with 3% hydrochloric acid for 40 min and then filtered again. The sample was subjected to a second decalcification step for 40 min, followed by liquid-phase transformation in an alkaline ethanol solution for another 40 min. Finally, dehydration with anhydrous ethanol yielded sodium alginate, which was then dried in an oven at 55 °C and ground into a powder [19].

The ethanol method involved adding 95% ethanol to the alkaline treatment solution to adjust the final ethanol concentration to 30% (*v*/*v*), followed by filtration. The sodium alginate precipitate was washed three times with 30% ethanol to eliminate any residual alkaline solution and then dehydrated with anhydrous ethanol, dried in an oven at 55 °C, and finally ground into a fine powder [20].

#### 2.3.3. Comparison of the Extraction Yields of Sodium Alginate from Different Parts of LN

**Figure 2:**
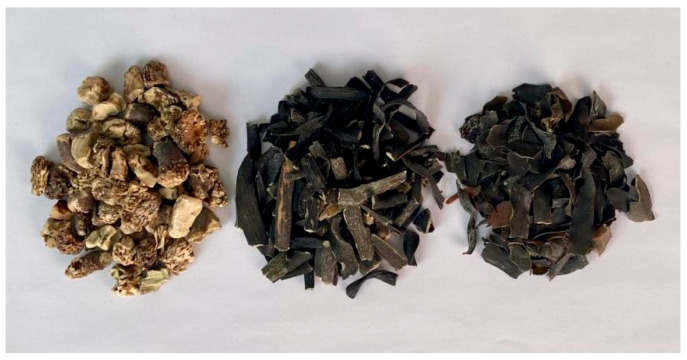
Holdfast, stipes, and blades of LN (from left to right).

The raw materials of LN were manually separated into holdfasts, stipes, and blades, as shown in Figure 2. In the whole LN, the blade accounts for approximately 60%, the stipe for approximately 25%, and the holdfast for approximately 15%. Based on the optimization results from the orthogonal experiments detailed in Section 2.3.1, alkaline treatment was performed on the holdfast, stipe, blade, and whole seaweed samples of LN. The alkaline treatment solutions obtained from the four raw materials were subjected to flocculation using three different methods: the ethanol, calcium, and acid methods. A total of 12 sodium alginate samples were obtained and labeled as follows: Alg-B (blade)-HCl, Alg-B-CaCl_2_, Alg-B-Ethanol, Alg-S (stipe)-HCl, Alg-S-CaCl_2_, Alg-S-Ethanol, Alg-H (holdfast)-HCl, Alg-H-CaCl_2_, Alg-H-Ethanol, Alg-W (whole seaweed)-HCl, Alg-W-CaCl_2_, and Alg-W-Ethanol. Subsequently, the extraction yields of sodium alginate were calculated and compared across these samples.

#### 2.3.4. Determination of Molecular Weight

The gel permeation chromatography (GPC) method developed by Pei [21] and Lim [22] was employed for the analysis. Gel permeation chromatography (GPC) coupled with a refractive index detector (RID) was employed under the following chromatographic conditions: a column temperature of 35 °C, a TSK-gel G6000 PWXL gel column, a flow rate of 0.5 mL/min, 0.1 mol/L NaCl as the eluent, and an injection volume of 20 μL. Calculations were based on the following equation:

$$\bar{M_{w,R}}\;=\;\sum_{i}W_{i}M_{i}\;=\;\frac{\sum H_{i}M_{i}}{\sum H_{i}}$$

where *W_i_*, *H_i_*, and *M_i_* are the mass fraction, height of the response value, and molecular weight of level i, respectively, and *M_i_* was obtained from the standard curve.

#### 2.3.5. Determination of M/G Values

The determination of M/G values in sodium alginate samples was conducted using high-performance liquid chromatography (HPLC) with the pre-column derivatization of PMP, based on the methods of Sun [23] and Chen [24]. Each of the twelve sodium alginate samples (10 mg) was treated with 0.5 mL of 80% H_2_SO_4_ and left at room temperature for 18 h. The H_2_SO_4_ solution was diluted to 2 mol/L and then heated in a boiling water bath for 5 h. After cooling the samples to room temperature, the pH was neutralized to 7.0 using CaCO_3_. The mixture was centrifuged to separate the supernatant, which was filtered and collected to obtain solutions containing mannuronic acid (M) and guluronic acid (G) derived from sodium alginate.

Standard solutions of mannuronic acid (M) and guluronic acid (G) were prepared at concentrations of 2.0, 1.0, 0.5, 0.25, and 0.125 mg/mL. Each standard solution (200 μL) was mixed with 200 μL of 0.3 mol/L NaOH, followed by thorough vortex mixing. Then, 240 μL of 0.5 mol/L PMP reagent was added, and the solution was vortexed until homogeneous, followed by heating at 70 °C for 1 h. Afterward, it was cooled to room temperature. Subsequently, 200 μL of 0.3 mol/L HCl was added to neutralize the solution. The mixture was extracted three times with chloroform, and the aqueous phase was collected, filtered through a 0.22 μm membrane filter, and prepared for HPLC analysis. The pre-column derivatization of sodium alginate samples was carried out as above. The chromatographic parameters were set as follows: a Zorbax SB-C18 column (150 mm × 4.6 mm, 5 μm) was utilized, with a mobile phase consisting of phosphate buffer (0.1 mol/L, pH = 6.8)/CH_3_CN (83/17, volume fraction); the flow rate was set at 0.8 mL/min; and the column temperature was maintained at 30 °C. An injection volume of 20 μL was employed, using a VWD detector, with the detection wavelength fixed at 245 nm.

#### 2.3.6. Infrared Spectroscopy

The method was modified based on the work of Roya [20] and Olga [25].

Each of the twelve sodium alginate samples (2 mg) was mixed with 180 mg of KBr, and the mixtures were dried to a constant weight. Each mixture was finely ground into a powder and compressed at a pressure of 16 MPa using a tablet press for 2 min to obtain a transparent pellet. The sample was analyzed by Fourier transform infrared (FTIR) spectroscopy to acquire its absorption spectrum. The test conditions included 64 scans, a resolution of 4 cm^−1^, and a spectral range of 500 to 4000 cm^−1^.

#### 2.3.7. Determination of Physical Properties of Sodium Alginate Samples

Viscosity was assessed using the method developed by Eduardo [26]. Moisture content and pH values were determined according to Sarah’s [27] methodology. Transmittance and water-insoluble substances were analyzed according to the procedures described by Yamashita [28] and Li [29].

#### 2.3.8. Statistical Analysis

Each experiment had three parallel data, and the results were processed in Excel and expressed as the mean ± standard deviation. Graphs were plotted using Origin 2019 and subjected to one-way ANOVA followed by Duncan’s test in SPSS 27.0. *p* < 0.05 was considered to indicate a significant difference, and *p* < 0.01 was considered to indicate a highly significant difference.

## 3. Results and Discussion

### 3.1. Optimal Process Determination

#### 3.1.1. Single-Factor Experiments on Alkaline Treatment Processes

**Figure 3:**
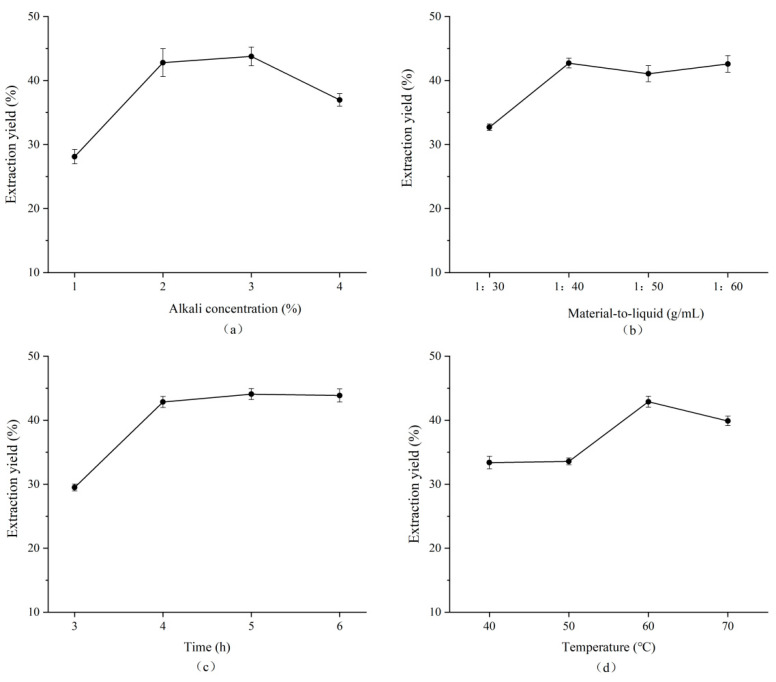
Effects of alkali concentration (**a**), material-to-liquid ratio (**b**), time (**c**), and temperature (**d**) on the yield of sodium alginate from Lessonia nigrescens (LN).

The effects of the alkali concentration, time, material-to-liquid ratio, and temperature on the alkaline treatment process are shown in Figure 3.

As the concentration of sodium carbonate increased, the content of uronic acid exhibited a trend of initially increasing followed by a decrease. This phenomenon was attributed to incomplete cell wall disruption at lower concentrations of sodium carbonate. Conversely, excessively high concentrations increased the pH of the alkaline treatment solution, leading to the degradation of sodium alginate and a decrease in uronic acid content. These findings are consistent with those reported by Mohammed [30], who investigated the effect of the Na_2_CO_3_ concentration on the extraction yield of sodium alginate. The study indicated that the optimal extraction efficiency was achieved at a sodium carbonate concentration of 5%. Beyond this point, further increases in the alkali concentration resulted in more than a 50% decline in the sodium alginate extraction yield.

As the time increased, the solubilization rate tended to rise and subsequently stabilized. However, excessively prolonged alkaline treatment led to the depolymerization of the sodium alginate, while the uronic acid content remained unchanged. Mazumder [31] also demonstrated that the time significantly influenced both the yield and quality of the sodium alginate; specifically, a shorter alkaline treatment duration resulted in decreased yields.

A low material-to-liquid ratio resulted in an overly viscous alkaline treatment solution, which hindered effective contact between the alkali and the cell walls of LN. As a result, the sodium alginate was incompletely dissolved. As the material-to-liquid ratio increased, however, the viscosity of the alkaline treatment solution decreased, thereby promoting a higher dissolution rate of sodium alginate. A similar trend was observed in the study conducted by Fawzy [32], who investigated the extraction yield of sodium alginate from *Sargassum* using varying alkali–algal ratios. Their findings indicated that the influence of the alkali–algal ratio on the sodium alginate extraction yield was comparable to that of the temperature and time. It was observed that when the alkali–algal ratio reached a certain peak value, further increases had no significant effect on the extraction yield of sodium alginate.

As the temperature increased, the rate of molecular movement was enhanced, leading to improved mass transfer efficiency. This facilitated the continuous dissolution of sodium alginate. However, upon reaching the optimal temperature, the extraction rate of sodium alginate began to decline due to its degradation at elevated temperatures, which ultimately reduced the extraction yield. Fertah [33] studied the extraction process of sodium alginate using Moroccan *Laminaria digitata* brown seaweed. Their findings indicated that both the surface area of the raw material and the temperature significantly affected the sodium alginate extraction yield. The maximum yield was observed at 40 °C; beyond this threshold, the extraction efficiency began to decline. Furthermore, under identical thermal conditions, an increased contact area between the raw materials and alkaline treatment solution was associated with a higher sodium alginate extraction yield.

#### 3.1.2. Results of Orthogonal Experimental Optimization of Alkaline Treatment Process

Table 2 summarizes the results of the orthogonal experimental design. Based on the range analysis, the influence of each factor on the sodium alginate extraction yield from LN was ranked as follows: D > A > B > C. The optimal combination identified was A_1_B_2_C_2_D_2_, achieving an extraction yield of 43.03%. To assess the stability of this optimal combination under actual production conditions, we repeated the experiments based on A_1_B_2_C_2_D_2_ three times. The resulting extraction yield was measured at 42.98 ± 0.08%, which was higher than those observed in other experimental groups listed in the table. This indicated that this optimal combination could consistently maintain a sodium alginate extraction yield of around 43%. Consequently, A_1_B_2_C_2_D_2_ was identified as the optimal alkaline treatment condition sequence for LN, with the process parameters including an alkaline treatment concentration of 2%, a duration of 4 h, a material-to-liquid ratio of 1:40, and a temperature of 60 °C.

### 3.2. Extraction Yield of Sodium Alginate

The optimization results from the orthogonal experiments described in Section 3.1.2 were utilized for the alkaline treatment of the holdfast, stipe, blade, and whole seaweed of LN. Subsequently, flocculation processes involving ethanol, calcium, and acid were performed on the alkaline treatment solutions derived from these four raw materials. The sodium alginate extraction yield was determined using a gravimetric method. As shown in Table 3, significant differences were observed among the extraction yields obtained by different methods for samples derived from the same part (*p* < 0.05). Specifically, under identical extraction conditions, the trend observed for the different flocculation methods regarding the sodium alginate yield was as follows: ethanol method > acid method > calcium method. This can be attributed to the direct use of ethanol in the ethanol method, which facilitates the efficient flocculation and precipitation of sodium alginate without the need for further transformations or substantial material loss. Conversely, the calcium method involved additional decalcification steps compared to the acid method. Prolonged decalcification might have led to the depolymerization of sodium alginate and consequently reduced its quality. Furthermore, findings from other scholars align with those presented in this study, showing that acid methods achieve higher extraction yields than calcium methods [10]. Due to the volatility and flammability of ethanol, its use in industrial production poses significant risks and should be approached with caution. The acid method demonstrated higher extraction efficiency than the calcium method, eliminating the need for a decalcification step. This not only reduces the production time but also lowers the associated costs. Therefore, the acid method is recommended for industrial application.

For the same flocculation method, significant differences in extraction yields were observed among different extraction parts (*p* < 0.05). The extraction yield followed the order stipe > blade > holdfast. Craigie [34] investigated the variations in alginate in brown algae based on the raw material parts, which aligned with the findings presented in this study.

### 3.3. Analysis of Molecular Weight Results

**Figure 4:**
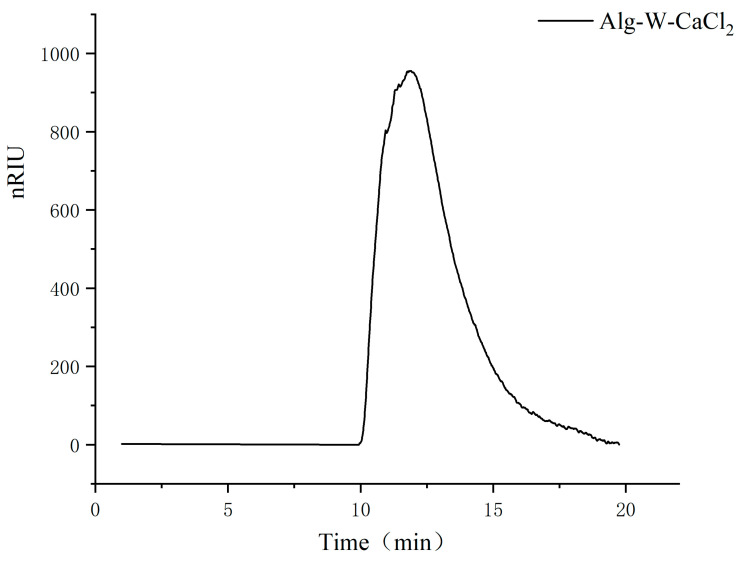
The GPC chromatogram of the sodium alginate sample Alg-W-CaCl_2_.

**Figure 5:**
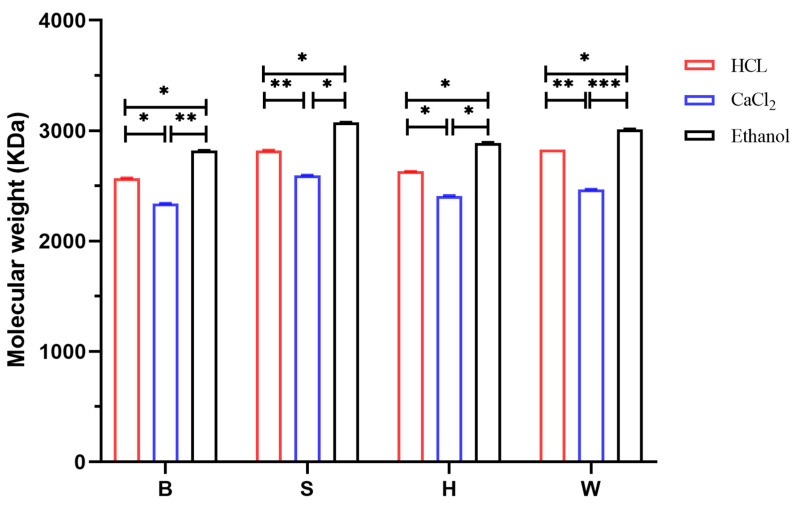
Relative molecular weights of sodium alginate in different parts under different methods. B (blade), S (stipe), H (holdfast), W (whole seaweed). “*” indicates *p* < 0.05; “**” indicates *p* < 0.01; “***” indicates *p* < 0.001.

Figure 4 shows the GPC chromatogram of the sodium alginate sample Alg-W-CaCl_2_. It can be observed from the figure that the elution time for the molecular weight was approximately between 10 and 14 min. The molecular weight distribution of the sodium alginate was relatively concentrated, with low content of high-molecular-weight impurities. The extraction process of sodium alginate demonstrated uniformity in terms of molecular weight distribution. The relative molecular weight of the sample could be calculated based on the peak area. Other samples exhibited the same trend as the sample Alg-W-CaCl_2_. Figure 5 shows the molecular weights of all twelve groups of sodium alginate samples, which ranged from 2343 to 3074 kDa. The ANOVA results revealed that all parts of LN exhibited highly significant differences in molecular weight among the three flocculation methods (*p* < 0.01), with statistically significant intergroup differences, indicating that the different flocculation methods had significant effects on the molecular masses of the samples. The ethanol flocculation method yielded sodium alginate samples with higher relative molecular weights, whereas the calcium flocculation method resulted in the lowest molecular weights among the various flocculation processes. This discrepancy could be attributed to the fact that the acids used during the calcium extraction process compromised the structural integrity of sodium alginate to some extent, while the ethanol method employed relatively mild extraction conditions. The molecular weight of sodium alginate plays a crucial role, as variations in molecular weight significantly affect the mechanical and thermal properties of alginate-based products [35]. The viscosity, mechanical properties, and other functional characteristics of sodium alginate samples were influenced by their molecular weights. Roya [36] studied a sodium alginate sample extracted using the high-voltage electrode discharge (HVED) method, which had a molecular weight of 1.19 × 10^5^ g/mol as determined by HPSEC coupled with MALLS. In comparison, the molecular weight of the sodium alginate extracted in this study was significantly higher.

### 3.4. Analysis of M/G Results

**Figure 6:**
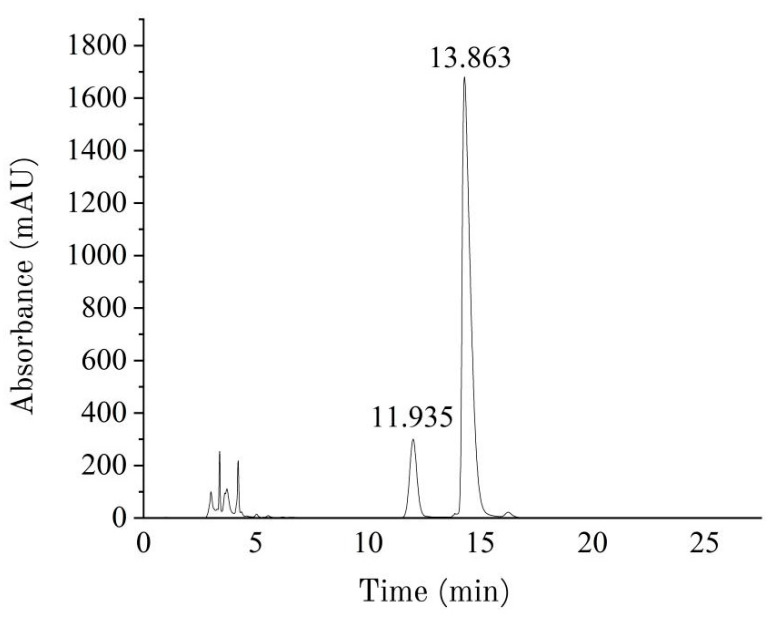
HPLC chromatogram of the sodium alginate sample Alg-S-CaCl_2_.

**Figure 7:**
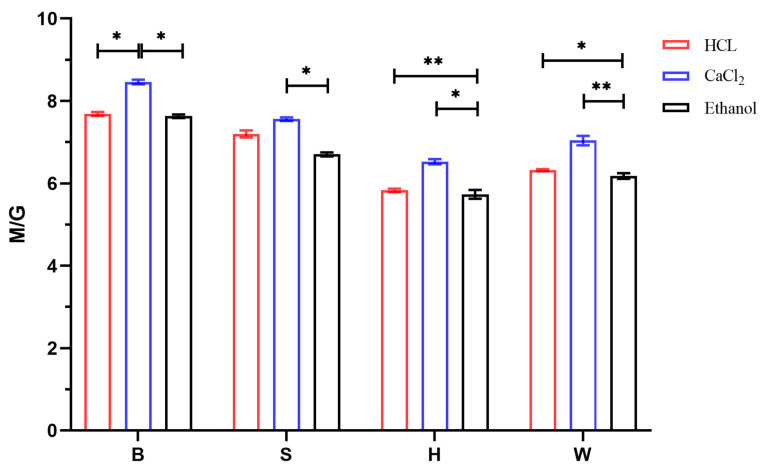
M/G of sodium alginate in different parts under different methods. B (blade), S (stipe), H (holdfast), W (whole seaweed). “*” indicates *p* < 0.05; “**” indicates *p* < 0.01.

Figure 6 shows the HPLC chromatogram of the sodium alginate sample Alg-S-CaCl_2_. The peak before 5 min is the PMP peak, the peak at 11.935 min is the elution time for G, and the peak at 13.863 min is the elution time for M. The figure indicates that the separation between M and G was good and highly effective, with minimal interference peaks other than that of the PMP reagent. It could thus be inferred that the acid hydrolysis of the sodium alginate sample was thorough, and no undegraded polymers were present. Figure 7 illustrates the M/G values of the twelve groups of sodium alginate samples. Based on these data, it could be concluded that the M/G values for these samples ranged from 5.73 to 8.46, which are notably higher than those of commercial sodium alginate products. Jiao [37] reported the M/G values for four commercial sodium alginate samples as follows: 5.70, 4.79, 6.01, and 3.97. The ANOVA results indicated that the calcium flocculation method yielded significantly higher M/G ratios than both the acid and ethanol methods across all algal parts (*p* < 0.05). However, the effects of the different flocculation methods on the sodium alginate M/G ratios varied depending on the algal part. The results indicated that LN exhibited a higher M/G ratio, which was attributed to the relatively greater content of M monomers compared to G monomers in the uronic acid composition of sodium alginate derived from the raw material used in this study. The M/G value in the blade portion was higher than those in both the stipe and holdfast parts, reaching a maximum of 8.46 for samples extracted using the calcium method. The extraction technique had a significant effect on the M/G values across different algal parts. Specifically, for samples obtained from identical parts of the raw material, the M/G values followed the order calcium method > acid method > ethanol method. Under consistent extraction conditions, there was a discernible trend regarding the M/G values among the extracted sodium alginates from various parts of LN: blade > stipe > holdfast. The variation in the M/G ratio across different parts of seaweed is primarily associated with their distinct functional roles. The blades, which are mainly responsible for photosynthesis, are richer in mannuronic acid (M), resulting in a higher M/G ratio. In contrast, the holdfasts and stipes, which serve a structural and supportive function, requiring greater mechanical strength, contain a higher proportion of guluronic acid (G), leading to a lower M/G ratio. Roya [15] found that differences in the M/G ratio significantly affected the rheological properties, chemical characteristics, and structural features of alginate. Jones’s [38] research indicated that the M/G ratio affects the physiological and rheological properties of alginate and that the physical and chemical characteristics of alginate vary according to the M and G content and their distribution.

### 3.5. Analysis of Infrared Spectroscopy Results

**Figure 8:**
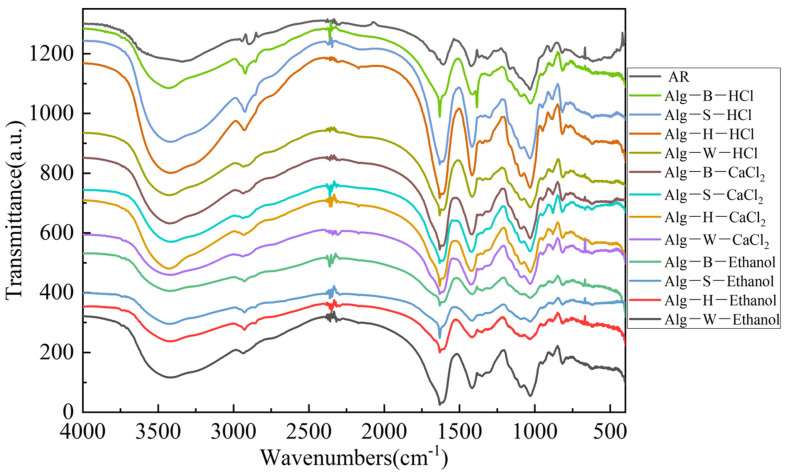
FTIR of sodium alginate in different parts under different methods.

Figure 8 illustrates the FTIR spectra of the twelve sodium alginate samples and a commercial sodium alginate standard (analytical reagent). Compared to the commercial sodium alginate, the positions of all absorption peaks were largely consistent. This observation indicates that the structural integrity of the functional groups was preserved throughout both the alkaline treatment and flocculation processes involving sodium alginate. In Figure 8, the peak at 3418 cm^−1^ can be attributed to O-H stretching vibrations, that at 2932 cm^−1^ to C-H stretching vibrations, and those at 1613 and 1415 cm^−1^ can be attributed to the asymmetric and symmetric stretching vibrations of the C=O bond in the -COOH group, respectively [39,40]. Additionally, an absorption peak observed at 1130 cm^−1^ was assigned to bending vibrations of C-C-H and O-C-H bonds within the pyran ring structure [41]. The characteristic peak at 1023 cm^−1^ corresponded to the stretching vibrations of the C-C bonds in the mannuronic acid (M) and guluronic acid (G) residues. The absorption band at 940 cm^−1^ was assigned to the stretching vibration of the C-O bond in the uronic acid residue. Additionally, mannuronic acid (M) was represented by a distinct absorption peak at 820 cm^−1^, while guluronic acid (G) was indicated by an absorption peak at 787 cm^−1^. Notably, the characteristic absorption peak for mannuronic acid (M) at 820 cm^−1^ was clearly evident across all groups of sodium alginate [42]. However, the characteristic absorption band of guluronic acid (G) at approximately 787 cm^−1^ was weak and poorly resolved, and infrared spectroscopy was only able to identify functional groups in the sodium alginate samples and not to determine the M/G ratio. Therefore, it was necessary to seek alternative methods for comparative microstructural analysis. Kyle [43] determined the M/G ratio of sodium alginate using ^1^H NMR analysis, not only obtaining the M/G value but also revealing the differences in alginate monads (M, G), dyads (MM, GG, MG), and triads (MGM, GGM). These differences were attributed to the variability of the biomass. Noelia [44] also noted that factors such as fluctuations in moisture content could reduce the precision of FTIR measurements of the M/G ratio and introduce interference. Meanwhile, FTIR spectroscopy has proven effective for the rapid identification of seaweed-derived polysaccharides, with applications spanning both food and non-food industries.

### 3.6. Analysis of Physical Properties of Sodium Alginate Samples

#### 3.6.1. Viscosity Result Analysis

**Figure 9:**
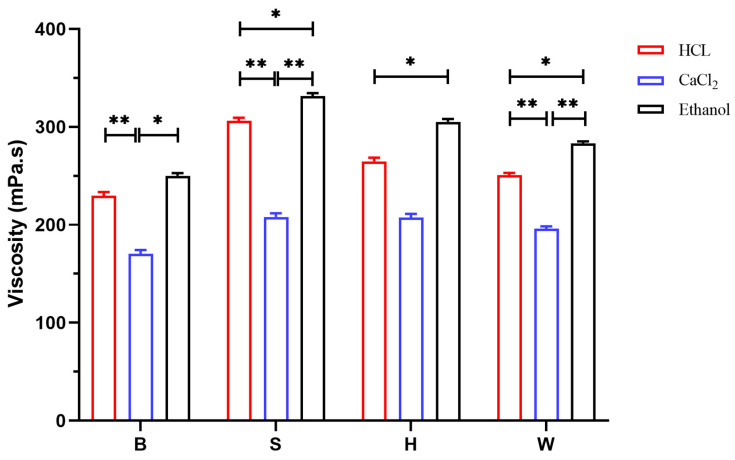
Viscosity of sodium alginate in different parts under different methods. B (blade), S (stipe), H (holdfast), W (whole seaweed). “*” indicates *p* < 0.05; “**” indicates *p* < 0.01.

The viscosity of the sodium alginate solution is a critical indicator in assessing the product quality of sodium alginate. The viscosity data for each sample are summarized in Figure 9. Among the three processing methods, the viscosity of the sodium alginate samples obtained via the ethanol method ranged from 250 to 331 mPa·s, whereas those derived from the acid method exhibited a viscosity range of 230 to 306 mPa·s. In contrast, the sodium alginate samples extracted using the calcium method exhibited a viscosity range of 170 to 208 mPa·s. The ANOVA results demonstrated that the viscosity of sodium alginate obtained using the ethanol method was significantly higher than that in both the acid and calcium methods (*p* < 0.05). While the acid method produced higher viscosity values compared to the calcium flocculation method, the difference was not statistically significant (*p* > 0.05). Notably, the sodium alginate samples extracted via different flocculation processes from LN stipe parts exhibited significant variability in viscosity. The most significant differences in viscosity were observed in samples obtained via the ethanol method, exhibiting significantly higher values than those extracted using the acid and calcium methods. This trend was consistently observed across other parts (blade, holdfast, and whole) of LN, where the effectiveness of the flocculation methods was ranked as follows: ethanol > acid > calcium. This phenomenon could be attributed to the flocculation environments employed in both the acid and calcium methods, which maintained pH levels below 4. Such acidic conditions lead to the disruption of chemical bonds within polymer chains, consequently diminishing the overall viscosity of sodium alginate. Furthermore, under identical extraction conditions, the source part significantly influenced the viscosity of the resulting sodium alginate, following the order stipe > holdfast > blade. This trend was consistently observed in both the calcium and ethanol flocculation methods. The findings regarding the effects of the flocculation method and raw material source on the viscosity of sodium alginate align with the results presented in Section 2.3 concerning the molecular weight; specifically, a higher molecular weight correlated with increased viscosity. Tatyana [45] indicated that the viscosity of sodium alginate solutions was strongly dependent on the molecular mass and that a reduction in molecular mass resulted in a significant reduction in viscosity. Andrea [46] investigated the influence of the molecular weight on the rheological properties of sodium alginate using rheological techniques and established a clear correlation between the viscosity and molecular mass. These findings demonstrate that sodium alginate with a higher molecular weight exhibits greater viscosity, which aligns with the results of the present study.

#### 3.6.2. Analysis of Results of Water-Insoluble Substance Determination

**Figure 10:**
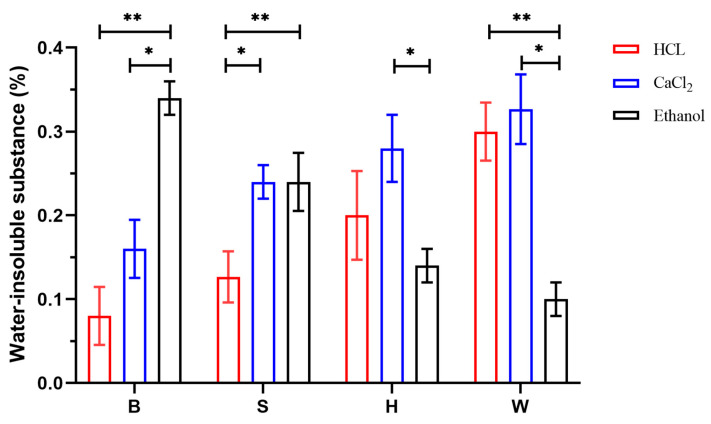
Water-insoluble content of sodium alginate in different parts under different methods. B (blade), S (stipe), H (holdfast), W (whole seaweed). “*” indicates *p* < 0.05; “**” indicates *p* < 0.01.

The water-insoluble matter present in sodium alginate primarily consisted of insoluble impurities that were encapsulated within the sodium alginate precipitate. This included impurities associated with the flocculation process, as well as those generated during subsequent reactions. The results regarding the water-insoluble matter for each group of sodium alginate samples are illustrated in Figure 10. As observed in the graph, there was notable similarity in the variations in water-insoluble matter content between the blade parts of sodium alginate extracts with higher concentrations and the stipe parts. Specifically, the water-insoluble matter content was highest in samples extracted using the ethanol and calcium methods and lowest in those obtained via the acid flocculation method. Conversely, holdfast-derived sodium alginate showed significantly higher content of water-insoluble matter when extracted using the calcium method. Moreover, only minor differences were observed in the water-insoluble matter content between the acid and calcium flocculation methods. However, the ethanol method yielded the smallest amount overall. The ANOVA revealed significant differences in water-insoluble matter content among the three flocculation methods across different algal parts (*p* < 0.05). Specifically, the acid and ethanol flocculation methods exhibited highly significant differences (*p* < 0.01) in the water-insoluble matter content of sodium alginate extracted from the L, S, and M parts. Meanwhile, the calcium and ethanol flocculation methods showed statistically significant differences (*p* < 0.05) in the water-insoluble matter content of sodium alginate. These results demonstrate that both the flocculation method and algal part have significant effects on the water-insoluble content of sodium alginate.

#### 3.6.3. Analysis of Other Physicochemical Properties

The results regarding the moisture content, pH, and transmittance of the 12 groups of sodium alginate samples extracted under varying process conditions are presented in Table 4. It was evident that all 12 sample groups exhibited a flocculent texture. Notably, sodium alginate extracted using the acid and calcium methods exhibited a light yellow color, whereas samples obtained via the ethanol method appeared light brown. The primary reason for this phenomenon was that the ethanol method relied solely on ethanol to precipitate sodium alginate from the alkaline treatment solution. Consequently, pigments and impurities in the alkaline solution were adsorbed and entrapped during this process, co-precipitating with sodium alginate and resulting in a darker sample color. In contrast, both the acid and calcium methods involved the use of hydrochloric acid, and the acidic conditions disrupted certain pigments and decomposed some impurities, thereby yielding products with a lighter color. The moisture content of the sodium alginate samples ranged from 5.54% and 7.10%; this moisture content complies with the FCC standards. The pH values ranged from a low of 7.33 to a high of 7.88. Furthermore, the transmittance of sodium alginate was excellent, with minimum and maximum values recorded at 97.57% and 99.77%, respectively, suggesting that the extracted sodium alginate samples possessed good solubility and a light color.

## 4. Conclusions

This study optimized the alkaline extraction of sodium alginate from *Lessonia nigrescens* (LN), determining the ideal conditions as a 2% alkali concentration, 4 h, a 1:40 material-to-liquid ratio, and 60 °C, achieving a high yield of 43.03%—significantly surpassing industrial standards (25–29%) [47]. Under identical flocculation methods, the sodium alginate extraction yield was highest from the stipe and lowest from the holdfast. When the extraction part was held constant, the ethanol-based method yielded the highest alginate recovery, whereas the calcium precipitation method resulted in the lowest yield. Ethanol-based extraction from the stipe yielded a superior molecular weight and viscosity due to milder processing, although cost and safety concerns make calcium-based methods favorable in industry.

The sodium alginate samples extracted from LN using the three methods were all of good quality and met the physicochemical requirements specified in the national standard GB 1886.243-2016 [48]. Their molecular weights (2343–3074 kDa), M/G ratios (5.73–8.46), and viscosities (170–331 mPa·s) varied depending on the flocculation method and algal part, with samples derived from the stipe exhibiting the highest quality. These findings support scalable, efficient production, offering a theoretical foundation for industrial applications. Future research should focus on large-scale implementation.

## Figures and Tables

**Table 1:** The factors and levels of the orthogonal test of LN.

Factor				
	A	B	C	D
Level	Alkali Concentration	Time	Material-to-Liquid Ratio	Temperature
	(%)	(h)	(g/mL)	(°C)
1	2	3	1:30	50
2	3	4	1:40	60
3	4	5	1:50	70

**Table 2:** Orthogonal experimental design and range analysis of results for LN.

		Factor				Metric
		A	B	C	D	
Number		Alkali Concentration	Time	Material-Liquid Ratio	Temperature	Extraction Yield
		(%)	(h)	(g/mL)	(°C)	(%)
1		2	3	1:30	50	36.87
2		2	4	1:40	60	43.03
3		2	5	1:50	70	37.91
4		3	3	1:40	70	37.30
5		3	4	1:50	50	32.72
6		3	5	1:30	60	33.40
7		4	3	1:50	60	41.89
8		4	4	1:30	70	40.93
9		4	5	1:40	50	33.61
	K1	117.81	116.06	111.20	103.20	
	K2	103.42	116.68	113.94	118.32	
	K3	116.43	104.92	112.52	116.14	
	k1	39.27	38.69	37.07	34.40	
	k2	34.47	38.89	37.98	39.44	
	k3	38.81	34.97	37.51	38.71	
	R	4.80	3.92	0.91	5.04	
Optimal solution		A1	B2	C2	D2	

Note: K is the accumulation of experimental data at the same level with the same factor, k is the average value of the experimental data at the same level with the same factor, and R represents the extreme value, $R=max\;k_{i}-min\;k_{i}$ (i = 1, 2, 3).

**Table 3:** Effects of different precipitation methods on LN product yield.

Sample Name	Part of LN	Sedimentation Method	Extraction Yield %
Alg-H-Ethanol	Holdfast	Ethanol	40.62 ± 0.52 ^de^
Alg-H-CaCl_2_	Holdfast	Calcium	38.50 ± 0.61 ^h^
Alg-H-HCl	Holdfast	Acid	39.43 ± 0.64 ^g^
Alg-S-Ethanol	Stipe	Ethanol	46.02 ± 0.59 ^a^
Alg-S-CaCl_2_	Stipe	Calcium	41.23 ± 0.77 ^d^
Alg-S-HCl	Stipe	Acid	43.96 ± 0.39 ^b^
Alg-B-Ethanol	Blade	Ethanol	44.77 ± 0.58 ^b^
Alg-B-CaCl_2_	Blade	Calcium	39.54 ± 0.63 ^fg^
Alg-B-HCl	Blade	Acid	40.08 ± 0.42 ^efg^
Alg-W-Ethanol	Whole Seaweed	Ethanol	42.96 ± 0.37 ^c^
Alg-W-CaCl_2_	Whole Seaweed	Calcium	39.84 ± 0.49 ^efg^
Alg-W-HCl	Whole Seaweed	Acid	40.51 ± 0.47 ^def^

Note: Between groups of data, the same letter indicates a non-significant difference (*p* > 0.05), and different letters indicate a significant difference (*p* < 0.05).

**Table 4:** Analysis of the physical and chemical properties of sodium alginate samples.

Sample Name	Color	State	pH	Moisture (%)	Transmittance (%)
Alg-B-HCl	Light yellow brown	Flocculent	7.49 ± 0.15 ^cde^	6.48 ± 0.20 ^bcd^	98.64 ± 0.22 ^b^
Alg-S-HCl	Light yellow brown	Flocculent	7.53 ± 0.10 ^cd^	5.90 ± 0.05 ^ef^	98.75 ± 0.10 ^b^
Alg-H-HCl	Light yellow brown	Flocculent	7.86 ± 0.11 ^a^	5.91 ± 0.28 ^ef^	98.71 ± 0.07 ^b^
Alg-W-HCl	Light yellow brown	Flocculent	7.33 ± 0.12 ^e^	6.65 ± 0.36 ^abcd^	99.71 ± 0.16 ^a^
Alg-B-CaCl_2_	Light yellow brown	Flocculent	7.64 ± 0.07 ^bc^	7.10 ± 0.29 ^a^	99.55 ± 0.23 ^a^
Alg-S-CaCl_2_	Light yellow brown	Flocculent	7.88 ± 0.06 ^a^	6.66 ± 0.27 ^abcd^	99.61 ± 0.10 ^a^
Alg-H-CaCl_2_	Light yellow brown	Flocculent	7.52 ± 0.10 ^cd^	6.81 ± 0.15 ^ab^	99.49 ± 0.18 ^a^
Alg-W-CaCl_2_	Light yellow brown	Flocculent	7.73 ± 0.11 ^ab^	5.54 ± 0.30 ^f^	99.77 ± 0.19 ^a^
Alg-B-Ethanol	Brown	Flocculent	7.87 ± 0.04 ^a^	6.30 ± 0.04 ^cde^	97.57 ± 0.24 ^c^
Alg-S-Ethanol	Brown	Flocculent	7.36 ± 0.05 ^de^	6.73 ± 0.37 ^abc^	98.54 ± 0.09 ^b^
Alg-H-Ethanol	Brown	Flocculent	7.39 ± 0.05 ^de^	5.79 ± 0.21 ^f^	98.56 ± 0.14 ^b^
Alg-W-Ethanol	Brown	Flocculent	7.86 ± 0.10 ^a^	6.24 ± 0.15 ^de^	97.61 ± 0.12 ^c^

Note: Between groups of data, the same letter indicates a non-significant difference (*p* > 0.05), and different letters indicate a significant difference (*p* < 0.05).

## Data Availability

The data presented in this study are available on request from the corresponding author. (The data are not publicly available due to privacy.)
